# Correction: Gross anatomy, histology and blood vessel topography of the alimentary canal of the Inland Bearded Dragon (*Pogona vitticeps*)

**DOI:** 10.1371/journal.pone.0307698

**Published:** 2024-07-18

**Authors:** Elisabeth Engelke, Christiane Pfarrer, Katharina Radelof, Michael Fehr, Karina A. Mathes

There is an error in the caption for Fig 4. Please see the complete, correct Fig 4 caption here.

**Fig 4:**
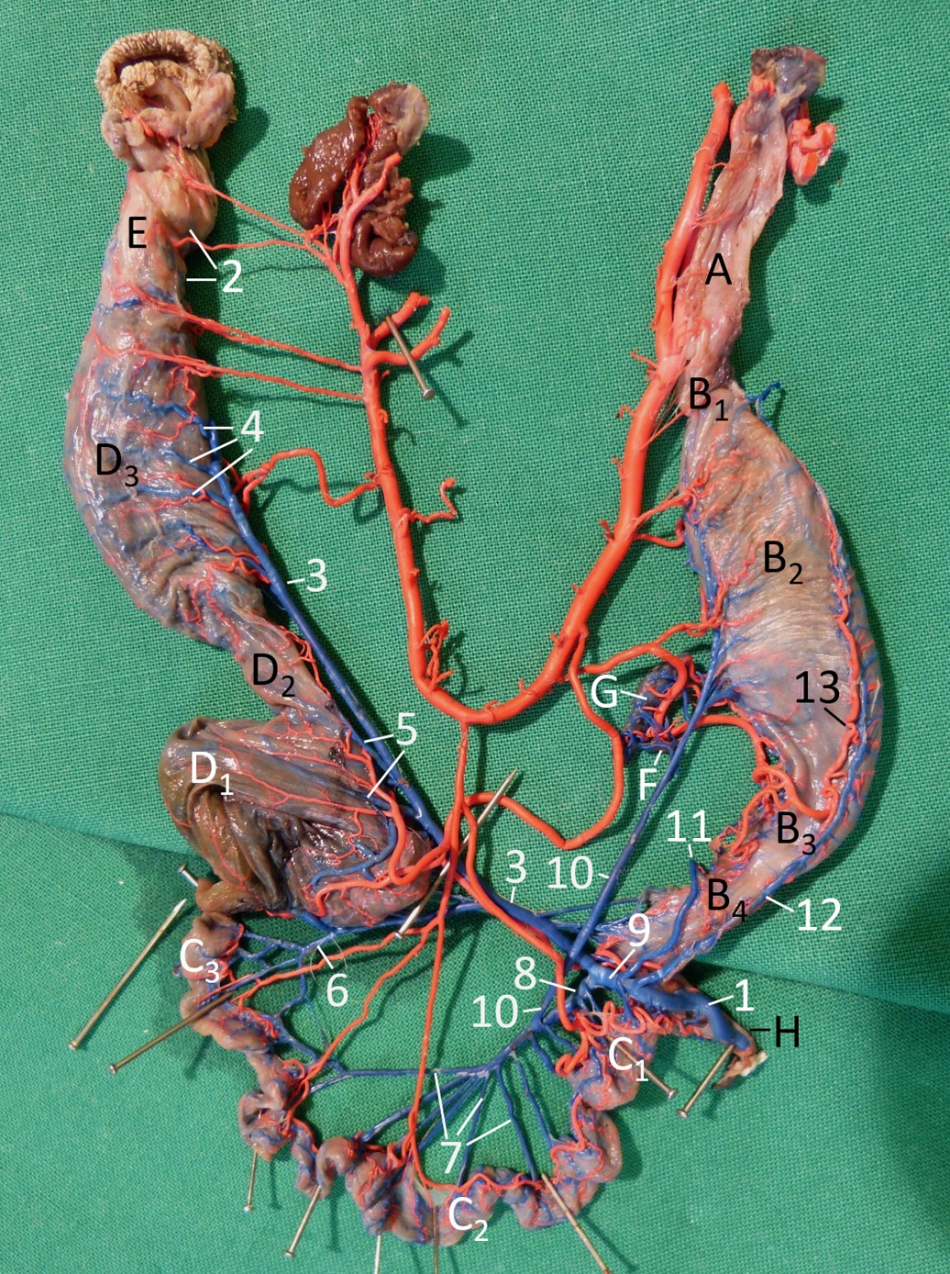
Vascularisation of the alimentary canal of a female Inland Bearded Dragon (*Pogona vitticeps*), exenterated, fixed with pins to be displayed single-planed, right aspect; arteries filled with red latex, veins filled with blue latex: veins labelled. A oesophagus; B1 cardiac part; B2 body of stomach; B3 pyloric part of stomach; B4 pylorus; C1 duodenum; C2 jejunum; C3 ileum; D1 colic ampulla; D2 colic isthmus; D3 rectum; E cloaca; F pancreas; G spleen; H bile duct. 1 portal vein; 2 cloacal veins; 3 caudal mesenteric vein; 4 rectal veins; 5 colic veins; 6 ileal vein; 7 jejunal veins, 8 cranial mesenteric vein; 9 common mesenteric vein; 10 left dorsal gastric vein; 11 right dorsal gastric vein; 12 ventral gastric vein; 13 ventral gastric artery.
