# Supplementary material for: Gross anatomy, histology and blood vessel topography of the alimentary canal of the Inland Bearded Dragon (Pogona vitticeps)
Source: PLoS One. 2020 Jun 23;15(6):e0234736. doi: 10.1371/journal.pone.0234736 (PMC7310682; doi:10.1371/journal.pone.0234736)
Supplement: Attachment — Submitted filename: Response_to_Reviewers.docx [file pone.0234736.s002.docx]

Response to reviewers

Manuscript ID PONE-D-19-31153:

"Gross anatomy, histology and blood vessel topography of the alimentary canal of the Inland Bearded Dragon (*Pogona vitticeps*)"

We would like to thank the reviewers for their thorough proof-reading of the manuscript and their thoughtful comments. We have considered each comment carefully and revised our manuscript to address the issues raised.

(The line numbers marked in yellow refer to the revised manuscript with track changes.)

Additional Editor Comments (if provided):

**Journal Requirements:**

Requirement #1.

*Please ensure that your manuscript meets PLOS ONE's style requirements, including those for file naming. The PLOS ONE style templates can be found at http://www.plosone.org/attachments/PLOSOne_formatting_sample_main_body.pdf and* [*http://www.plosone.org/attachments/PLOSOne_formatting_sample_title_authors_affiliations.pdf*](http://www.plosone.org/attachments/PLOSOne_formatting_sample_title_authors_affiliations.pdf)

We inserted continuous line numbers.

First page: We removed the “and” between the second last and last author, line 8. as the template for the first page does not show it.

File naming: We changed the captions of the figures composed of several images (Figs 2, 5, 6, 7) by removing the letters from the figure labels in the caption, so that the figure file names and the figure label in the caption are the same now. Fig 2: page 8, line 185; Fig 5: page 14, line 339; Fig 6: page 15, line 357; Fig 7: page 15, line 369.

Requirement #2.

*Please clarify the source of the animal cadavers used in this study. If they were pets, please state whether you received permission from the owners. Please also provide a reference for the source if relevant.*

We inserted the information that we asked the owners of the pets for permission into the Material and method section of the manuscript, page 4, lines 72-73.

Requirement #3.

*We note that you have provided funding information that is not currently declared in your Funding Statement. However, funding information should not appear in the Acknowledgments section or other areas of your manuscript. We will only publish funding information present in the Funding Statement section of the online submission form.*

*Please remove any funding-related text from the manuscript…*

We removed the funding information from the Acknowledgements section of the manuscript, page 27, lines 641-643.

*… and let us know how you would like to update your Funding Statement.*

Please remove "The author(s) received no specific funding for this work." from the Funding Statement section and insert instead " This publication was supported by Deutsche Forschungsgemeinschaft (https://www.dfg.de) and University of Veterinary Medicine Hannover, Foundation (https://www.tiho-hannover.de) within the funding programme Open Access Publishing. The funders had no role in study design, data collection and analysis, decision to publish, or preparation of the manuscript."

Requirement #4.

*Your ethics statement must appear in the Methods section of your manuscript. If your ethics statement is written in any section besides the Methods, please move it to the Methods section and delete it from any other section. Please also ensure that your ethics statement is included in your manuscript, as the ethics section of your online submission will not be published alongside your manuscript.*

We inserted our ethics statement into the Material and methods section of our manuscript, page 5, lines 99-103.

**Reviewer #1:**

Attached copy of the manuscript with comments

Comments:

Comment #1

*Page 3, line 50: Does this statistic apply to all lizards or just bearded dragons?*

The statistic applies to all examined lizards in the respective study [2].

Comment #2

*Page 5, line 110: beautiful specimen preparations and i like the intuitive labels for the arteries.*

Thank you! We enjoyed reading this comment!

Comment #3

*Page 5, line 115: I would love to see a figure showing this.*

As we did not receive an answer to our question to the editor about the permission to add figures until 24. April 2020, we did not add macroscopic figures.

Comment #4

*Page 5, line 117: I think you mean "L", correct?*

“L” is correct, we changed the letter, page 6, line 124.

Comment #5

*Page 6, line 131: I would suggest using "Where" instead of "When"*

We followed the suggestion of the reviewer and inserted “Where” instead of “When”, page 6, line 138.

Comment #6

*Page 6, lines 133-136: I would love to see a photo of this included.*

As we did not receive an answer to our question to the editor about the permission to add figures until 24. April 2020, we did not add macroscopic figures.

Comment #7

*Page 8, lines 172-173: It would be good to have a figure showing the parts of the cloaca. I have seen very few good dissection images of the three parts.*

As we did not receive an answer to our question to the editor about the permission to add figures until 24. April 2020, we did not add macroscopic figures.

Comment #8

*Page 8, line 185: add "of stomach"*

Following the Reviewer’s suggestion, we added “of stomach”, page 8, line 193.

Comment #9

*Page 9, line 220: remove comma*

We followed the suggestion of Reviewer 1, page 10, line 228.

Comment #10

*Page 11, lines 249-270: I would strongly suggest rewriting this section on veins to reflect the fact that veins are named where they drain a target organ and then come together to form larger veins instead of branching to supply individual organs as arteries do. We try to stress this difference to the students and it would be good to see it emphasized in the literature.*

According to the Reviewer’s suggestion, we removed the original text and re-wrote the description of the veins in the order of the blood flow, pages 11-13.

While writing the new text, we recognized that the dorsal gastric veins would be described in a better way, when we add “dorsal” to their term. Therefore, we added “dorsal” to the labelling of these veins in Fig 4, page 14, 327-328.

Comment #11

*Page 13, line 311-316: The histology figures are excellent. I would also like to see some higher res images of the smaller individual cells and structures discussed in the text if possible as well. I understand if this is not possible due to figure constraints, however.*

Following the suggestion of the Reviewer, we inserted one higher-resolution image to Fig 6 and two higher-resolution images to Figs 5 and 7, respectively. We adapted the figure captions of the Figs 5, 6 and 7 to the changes made, pages 14-16. The figure references in the text were also adapted, page 16, line 379-390.

Inserting the higher-resolution images, we recognized that the clearly visible brush border of the oesophagus was not yet mentioned in the manuscript. Thus, we inserted a sentence describing the oesophageal brush border, page 16, lines 380-381.

Comment #12 and #13

*Page 14, lines 328-329: delete	add "to be discerned" here*

We changed the sentence in the suggested manner, page 16, lines 389-390.

Comment #14

*Page 14, line 347-348: Figure?*

We added an indication of the brush border to Fig 6D, Fig 7E-F and a figure reference to the text, page 17, line 408-409.

Comment #15

*Page 15, line 355: Should there be a figure reference here? Fig 6A-C/4-4' perhaps?*

The Reviewer is right; there should have been a figure reference. We inserted the respective reference, page 17, lines 416-417.

Comment #16

*Page 16, line 383: change to "as well"*

We changed the wording in the suggested manner, page 18, line 444.

Comment #17

*Page 22, line 544: add "also" here*

We changed the wording in the suggested manner, page 24, line 605.

**Reviewer #2:**

Comment #1

*Line 108. Change the label of the testicle in the gross image. It is labeled “K”. I would switch the liver and testicle letters. Many practitioners easily forget that the kidneys in bearded dragons are within the pelvis. If they glance at the image, they will immediately think K=Kidney.*

We followed the suggestion of the reviewer and switched the letters indicating liver and testicle in Figure 1, the text (page 6, line 125) and the figure caption (page 5, line 115).

Comment #2

*Line 139: If there are only two lobes of the pancreas, this should be noted.*

There is also a pancreatic body between the two lobes. We inserted a sentence explaining this fact, page 7, lines 148-150.

Comment #3

*Line 142: Is the spleen at the cranial end of the dorsal or ventral pancreatic lobe.*

The spleen is located at the dorsal lobe of the pancreas. We added this detail to the respective sentence, page 7, lines 150-151.

Comment #4

*Line 150 to 157 and 167. Are there approximate measurements to these ‘larger, smaller, longer’ descriptions? I realize this might not be possible given the range in size of the females. The male specimens seem to be approximately the same size. Or maybe there is some way to give the reader a guesstimate: ‘the jejunum was about twice as long as the duodenum’. ‘The duodenum is ¼ larger in diameter than the jejunum.’ You do provide a measurement for the caecal diverticulum.*

Exact measurements of the specific sections of the alimentary canal were taken and have already been published by our work group in PLOS ONE [6]. To give the reader the information where to find the measurement values, we added an indication with the reference to Material and methods, page 4, lines 88-89.

Comment #5

*260 to 261: I want to make sure I understood this….the ‘smaller veins from the’ or the ‘further large vein left’ of the ‘cranial mesenteric vein’ drains the pancreas (both lobes?) and the spleen? I am unclear about ‘this branch’ arose form the caudal mesenteric vein (in two out of seven animals).*

We re-wrote the chapter about the veins according the suggestion of reviewer 1. In doing so, we explained that it is the left dorsal gastric vein draining the pancreas and spleen, page 13, lines 309-311.

We added “the dorsal lobe of” to “the pancreas”. page 13, line 310

Comment #6

*327 and 563: Ventriculus? Do bearded dragons have a ventriculus? Is there a reference for this? I thought this was only in crocodiles and alligators.*

Checking the text of our manuscript, we became aware of the fact that we did not use any other Latin term for other organs or stuctures. Thus, we removed the Latin term “ventriculus” from the text, page 16, line 388 and page 25, line 625.

The term “ventriculus” is the Latin anatomic term for the single-chambered stomach in veterinary medicine [7]. In birds and crocodiles/alligators, there is an additional gastric chamber, the proventriculus, which is located cranially to the “main” stomach, the ventriculus. In birds, the proventriculus is also called glandular stomach, and the ventriculus is also known as muscular stomach or gizzard.

Again, the authors are grateful for the very helpful editorial suggestions and improving remarks.
